# The Biological Significance and Implications of Planar Cell Polarity for Nephrology

**DOI:** 10.3389/fphys.2021.599529

**Published:** 2021-02-26

**Authors:** Eugenia Papakrivopoulou, Daniyal J. Jafree, Charlotte H. Dean, David A. Long

**Affiliations:** ^1^Developmental Biology and Cancer Programme, UCL Great Ormond Street Institute of Child Health, London, United Kingdom; ^2^Department of Internal Medicine and Nephrology, Clinique Saint Jean, Brussels, Belgium; ^3^UCL MB/Ph.D. Programme, Faculty of Medical Science, University College London, London, United Kingdom; ^4^National Heart and Lung Institute, Imperial College London, London, United Kingdom

**Keywords:** cytoskeleton, extracellular matrix, glomerular disease, kidney development, polarity, polycystic kidneys

## Abstract

The orientation of cells in two-dimensional and three-dimensional space underpins how the kidney develops and responds to disease. The process by which cells orientate themselves within the plane of a tissue is termed planar cell polarity. In this Review, we discuss how planar cell polarity and the proteins that underpin it govern kidney organogenesis and pathology. The importance of planar cell polarity and its constituent proteins in multiple facets of kidney development is emphasised, including ureteric bud branching, tubular morphogenesis and nephron maturation. An overview is given of the relevance of planar cell polarity and its proteins for inherited human renal diseases, including congenital malformations with unknown aetiology and polycystic kidney disease. Finally, recent work is described outlining the influence of planar cell polarity proteins on glomerular diseases and highlight how this fundamental pathway could yield a new treatment paradigm for nephrology.

## Introduction

The generation of normal adult renal structure and function is not only defined by the diversity of cell types within the kidney, but also the orientation of these cells in two-dimensional and three-dimensional space. The correct orientation of cells is required for the mechanical and molecular interactions that dictate how organs develop in the embryo, their physiological functions and also their response to injury. A critical determinant of these processes is cell polarity, the organised establishment of asymmetry in the shape or molecular profile of cells or groups of cells.

**FIGURE 1:**
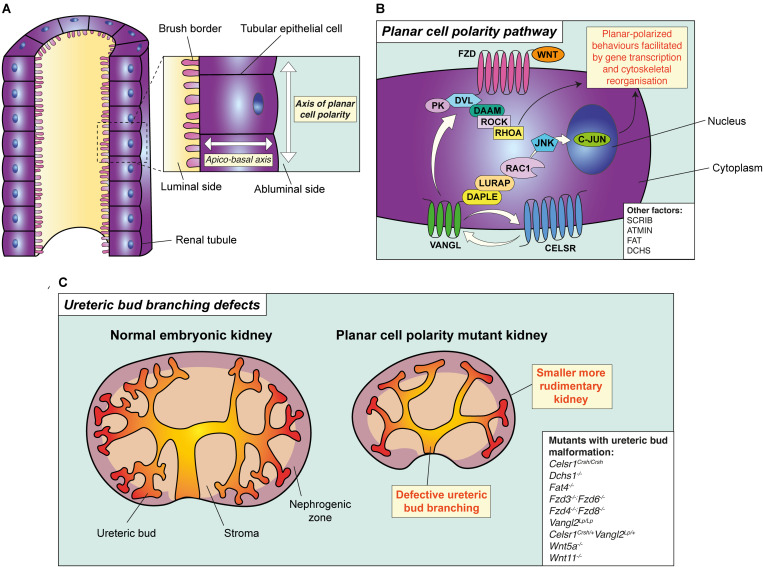
The principle and pathway of planar cell polarity and its role in branching morphogenesis. **(A)** The principle of planar cell polarity (PCP). An individual tubular epithelial cell is shown from a tubule cut lengthways. The axis formed between the luminal side of the cell and the abluminal side is termed the apico-basal axis. Conversely, the axis of orientation within the plane of the tubule is the axis of PCP. A key feature of PCP is the asymmetric expression of core PCP proteins, such as VANGL and FZD, setting the direction of polarity across the plane of the tubule. **(B)** The planar cell polarity pathway and its proteins, as demonstrated in a tubular epithelial cell. Binding of WNT ligands (WNT5A or WNT11) to FZD receptors leads to the recruitment of DVL proteins to the membrane resulting in the formation of a multi-protein complex. This complex interacts with multiple effector molecules. Downstream of DVL, two independent and parallel pathways have been proposed. The first pathway signals to RHOA through the formin homology protein; DAAM1, and ROCK. The second pathway involves the adaptor proteins DAPLE and LURAP, ultimately resulting in C-JUN-dependent transcription and coordinating planar-polarised behaviours. Other factors involved in PCP are shown in the white box. **(C)** PCP and ureteric bud branching. Mutation or deletion of core PCP proteins in mouse lead to the simplification of ureteric bud branching, particularly in the caudal component of the organ, accompanied by a reduction in kidney size. All mouse models with disruption of PCP proteins which have also been demonstrated to have defective ureteric bud branching defects are shown in the white box.

There are two forms of cell polarity. Using the renal tubule as an example (Figure 1A), the first form is apical-basal polarity. Apico-basal polarity is dictated by the asymmetric distribution of proteins, organelles and the cytoskeleton between luminal and abluminal sides of tubular epithelial cells (Riga et al., 2019). The second form of cell polarity, and the focus of this Review, refers to the orientation of adjacent tubular epithelial cells perpendicular to the apico-basal axis. This form of polarity, within the two-dimensional plane of the tubule, is termed planar cell polarity (PCP) and controls multiple cellular behaviours collectively referred to as planar-polarised behaviours. PCP is required for early events during embryonic development such as the proper layout of the embryonic body plan (Gray et al., 2011; Roszko et al., 2015) and neural tube closure (Nikolopoulou et al., 2017; Butler and Wallingford, 2018). Later in development, PCP is key in orchestrating the formation of functional organs (Henderson et al., 2018). More recently, roles for PCP are emerging in postnatal contexts including cancer, wound healing and lung disease (Findlay et al., 2016; Daulat and Borg, 2017; Poobalasingam et al., 2017).

In this Review, we outline the relevance of PCP for nephrology. We first provide an overview of the proteins responsible for PCP and how it is regulated. We then highlight the diverse roles for PCP proteins in multiple facets of normal kidney development; discussing the evidence for planar-polarised behaviours or other functions of PCP proteins in each context. Finally, we discuss recent evidence implicating PCP proteins in inherited and acquired forms of kidney disease, including congenital malformations of the kidney and urinary tract (CAKUT), polycystic kidney disease (PKD), and glomerular diseases.

## A Brief Overview of Planar Cell Polarity

### Molecules Involved in Planar Cell Polarity

The signalling molecules governing PCP were first discovered by screening for genes responsible for the uniform orientation of hairs on the wing and abdomen of the fruit fly (Simons and Mlodzik, 2008). These experiments identified a set of evolutionarily conserved proteins constituting the “core” PCP pathway (reviewed in Simons and Mlodzik, 2008; Gray et al., 2011; Davey and Moens, 2017; Butler and Wallingford, 2018). Transmembrane receptors, including Cadherin EGF LAG seven-pass G-type receptor (CELSR), Van Gogh (VANGL) or Frizzled (FZD) (Table 1) transmit information on the polarity of a cell to its neighbours (Butler and Wallingford, 2017). These receptors, once stimulated, signal to cytoplasmic proteins such as Dishevelled (DVL) or Prickle (PK) which activate downstream effector molecules (Davey and Moens, 2017) and drive cytoskeletal alterations within the cell. In this way, polarity cues are propagated to influence cell shape and behaviour (Figure 1B; Winter et al., 2001; Yasunaga et al., 2015). A characteristic feature of the core PCP pathway is the accumulation of different proteins, such as VANGL and FZD, on opposing sides of the same cell relative to the plane of the tissue (Zallen, 2007); setting the direction of planar polarity. Molecular asymmetry of PCP proteins is maintained across individual cells within polarised tissues by cell-cell contacts. Cell-cell contact facilitates signalling complexes between PCP proteins on adjacent cells; allowing these cells to relay polarity cues to one another within a tissue (Butler and Wallingford, 2017).

**TABLE 1:** Summary of kidney phenotypes in mice with mutations or knockouts of core PCP protein-encoding genes.

Gene	Function in PCP and localisation in kidney	Phenotype in mutant or knockout kidneys	Evidence for PCP in these contexts
*Celsr*	Encodes a family of seven-pass transmembrane proteins localised on membranes on the opposite side of planar-polarised cells to FZD. Permits exchange of polarity information between cells. *Celsr1-3* expressed on ureteric bud epithelium at E14.5 (Shima et al., 2002). *Celsr1* expressed by S-shaped bodies, podocytes, and proximal tubular epithelium at E18.5 (Yates et al., 2010)	*Celsr1^*Crsh/*+^; Vangl2^*Lp/*+^*: more rudimentary ureteric trees than single heterozygotes defects and more prominent in caudal region, tubular dilations not observed, immature glomeruli (Brzóska et al., 2016) *Celsr1^*Crsh/Crsh*^*: reduced ureteric bud tip number more prominently in caudal portion of the kidney, cortical tubular dilation, no glomerular phenotype (Brzóska et al., 2016)	Deviation of axis of mitotic spindles in tubular epithelial cells in *Celsr1^*Crsh/Crsh*^* mice relative to longitudinal axis of tubule (Brzóska et al., 2016), suggestive of defective orientation of cell division. Phenotypes from double heterozygous *Celsr1^*Crsh/*+^; Vangl2^*Lp/*+^* animals (Brzóska et al., 2016) suggest these components interact and potentially affect the same biological processes. Other planar-polarised behaviours or asymmetric expression of CELSR1 not reported during ureteric bud branching or glomerular morphogenesis
*Fzd*	Encodes G protein-coupled receptors which accumulate on opposite side of cell membrane to VANGL/CELSR in planar-polarised cells. Respond to WNT ligands to align planar-polarised tissues. FZD3 and FZD6 expressed by proximal tubular and collecting duct epithelial cells at E18.5 (Kunimoto et al., 2017)	*Fzd3^–/–^; Fzd6^–/–^*: dilation of proximal tubules and collecting ducts (Kunimoto et al., 2017) *Fzd4^–/–^; Fzd8^–/–^*: reduced ureteric bud tip number (Ye et al., 2011)	FZD3 and FZD6 asymmetrically distributed on the distal side of proximal tubular and collecting duct epithelial cells (Kunimoto et al., 2017). Asymmetric distribution of FZD4 and FZD8 not ascertained. Increased number of epithelial cells in cross-sections of proximal tubules and collecting ducts of *Fzd3^–/–^;Fzd6^–/–^* embryonic kidneys (Kunimoto et al., 2017)
*Vangl*	Encodes proteins with four transmembrane domains which accumulate on the opposite side of cell membrane to FZD in planar-polarised cells. Permits exchange of polarity information between cells. VANGL1 and VANGL2 expressed by collecting duct epithelial cells at E18.5 (Kunimoto et al., 2017) and VANGL2 expressed by developing podocytes at E17.5 (Rocque et al., 2015).	*Vangl2^*Lp/Lp*^*: reduced ureteric bud tip number, immature glomeruli, proximal tubular, and collecting duct dilation (Yates et al., 2010; Kunimoto et al., 2017; Derish et al., 2020)	VANGL1 and VANGL2 asymmetrically localised on collecting duct epithelial cell membranes (Kunimoto et al., 2017) but asymmetry not clearly demonstrated in podocytes. Increased number of epithelial cells in cross-sections of proximal tubules or collecting ducts of *Vangl2^*Lp/Lp*^* (Kunimoto et al., 2017; Derish et al., 2020), *Vangl2^Δ^^*Ex*4/Δ^^*Ex*4^* and *Hoxb7-Cre;Vangl2^Δ^^*Ex*4/fl^* (Derish et al., 2020) embryonic kidneys. Changes in mitotic orientation not examined in the context of reduced podocyte number.
		Vangl2^Δ^^*Ex*4/Δ^^*Ex*4^*: dilation of collecting ducts (Derish et al., 2020)	
		*Celsr1^*Crsh/*+^; Vangl2^*Lp/*+^*: as above (Brzóska et al., 2016)	
		*Ksp-Cre;Vangl1^*fl/fl*^;Vangl2^*fl/fl*^*: dilation of collecting ducts (Kunimoto et al., 2017)	
		*Hoxb7-Cre;Vangl2^Δ^^*Ex*4/fl^* *: dilation of collecting ducts (Derish et al., 2020)	
		*Nphs2-Cre:Vangl2^Δ^^*Ex*4/fl^* *: immature glomeruli and reduced podocyte (Rocque et al., 2015)	
		*Nphs2-Cre:Vangl2^*fl/fl*^*: immature glomeruli (Papakrivopoulou et al., 2018)	

*^∗^Vangl2 ^ΔEx4^ allele generated by crossing Vangl2^fl/fl^ mouse with MORE-Cre to delete exon 4 of Vangl2 in all epiblast cells.*

The Fat (Ft)-Dachsous (Ds)-Four-jointed (Fj) module is another pathway identified in fruit fly which has been shown to regulate PCP (reviewed in Matis and Axelrod, 2013). There is also some evidence, from genetic knockout studies, that the mouse homologues of this pathway may interact with core PCP proteins to coordinate planar-polarised behaviours in mammalian systems (Saburi et al., 2008, 2012). However, asymmetric expression of the mammalian homologues of the Ft-Ds-Fj module is yet to be demonstrated (Devenport, 2014).

### Extracellular and Intracellular Control of Planar Cell Polarity

Multiple extracellular and intracellular cues coordinate planar-polarised behaviours *via* direct or indirect activity on PCP proteins. Extracellular cues include biochemical stimuli, such as the PCP-associated WNT ligands WNT5A (Qian et al., 2007) or WNT11 (Chu and Sokol, 2016), which can appear in gradients across polarised tissues and influence PCP signalling by binding FZD receptors on cells (Habas et al., 2003; Gao et al., 2011, 2018; Chu and Sokol, 2016). Extracellular mechanical forces, generated by tissue growth (Aw et al., 2016) or fluid flow (Guirao et al., 2010), also influence and maintain planar polarity. Within cells, PCP proteins are subject to post-translational modification such as ubiquitinylation (Narimatsu et al., 2009; Cho et al., 2015) or phosphorylation (Shrestha et al., 2015; Kelly et al., 2016). These modifications in, addition to trafficking by microtubules (Shimada et al., 2006; Matis et al., 2014) or endocytosis (Devenport et al., 2011; Heck and Devenport, 2017) lead to the stabilisation, internalisation, redistribution, or degradation of PCP proteins to establish molecular asymmetry. Additionally, several intracellular molecules, such as dishevelled-associated activator of morphogenesis (DAAM) 1 and 2 (Habas et al., 2001), Scribble (SCRIB) (Courbard et al., 2009), and ATM interactor (ATMIN) (Goggolidou et al., 2014) interact with core PCP proteins to modulate planar-polarised behaviours. Thus, PCP is a dynamic process, whereby extracellular cues globally align cells in a tissue plane and intracellular cues fine-tune the responsiveness of individual cells to polarisation. Whether other factors characteristic of the microenvironment within the kidney, such as pH gradients, hypoxia or osmolality, influence planar-polarised behaviours or PCP proteins has not been explored.

## Planar Cell Polarity Orchestrates Kidney Organogenesis

PCP signalling elicits a variety of planar-polarised behaviours during the formation of organs, including orienting the axis of cell division (Gong et al., 2004; Ségalen et al., 2010; Li et al., 2017). During embryonic development, oriented cell division facilitates the segregation of cells during stem cell differentiation (Segalen and Bellaïche, 2009) and drives the elongation of tissues (Keller, 2006). PCP is also important in orchestrating collective and stereotypical movements of cells (Carmona-Fontaine et al., 2008; Tada and Heisenberg, 2012; Stramer and Mayor, 2017). The best studied collective cell movement governed by PCP is convergent extension, during which groups of cells narrow and intercalate along one axis and extend in the other (Tada and Heisenberg, 2012). Convergent extension results in tissue elongation (Huebner and Wallingford, 2018) and is a prominent feature of early embryogenesis and later organ formation (Ybot-Gonzalez et al., 2007; Chacon-Heszele et al., 2012).

Given these diverse functions, it is unsurprising that PCP signalling orchestrates several developmental phenomena during organogenesis of the kidney. The adult kidney originates from a primitive group of cells in the early embryo: the intermediate mesoderm. This population of cells divides, moves and eventually differentiates into a plethora of epithelial, endothelial and interstitial cell-types (Park et al., 2018; Combes et al., 2019) that together form the complex structure of the mature kidney. Genetic knockout or mutation of PCP protein-encoding genes, predominantly in mouse (Table 1), has provided evidence for roles of PCP proteins during ureteric bud branching, tubular morphogenesis and podocyte maturation. A key limitation of these experiments is the challenge of distinguishing underpinning cellular mechanisms. Alongside their role in planar-polarised behaviours, PCP proteins may have alternative functions, such as the spatial organisation of the extracellular matrix (ECM) through regulation of matrix metalloproteinase (MMP) activity (Williams et al., 2012; Dohn et al., 2013; Jessen and Jessen, 2017). Thus, to identify a planar-polarised behaviour is responsible for a given process in kidney development, it is important to experimentally ascertain whether molecular asymmetry of PCP proteins is a feature of each process studied, and that this asymmetry is lost upon genetic perturbation of PCP signalling.

### Planar Cell Polarity and Ureteric Bud Branching

The generation of the urinary collecting system is a highly dynamic process, beginning when the ureteric bud epithelia invades a “metanephric” mesenchymal population of cells near the tail-end of the embryo. Signals from the metanephric mesenchyme cause the ureteric bud to branch, with each branch terminating with a tip. Each tip is capped by condensing mesenchyme: the cap mesenchyme, which gives rise to all segments of the nephron. The ureteric bud branches stereotypically in a process termed branching morphogenesis; forming ∼3,000 tips by the end of mouse kidney development (Short and Smyth, 2020) and generating the renal pelvis and collecting duct network.

During early renal development, *Celsr1-3* transcripts have been localised to the ureteric bud by *in situ hybridisation* (Shima et al., 2002). Knock-in β-galactosidase reporters have also shown the ureteric bud to express *Fzd4* and *Fzd8* (Ye et al., 2011). Disruption of core PCP proteins in mice (Table 1) results in defective ureteric bud branching during kidney development, which manifests as a reduced number of ureteric bud tips (Figure 1C). For example, homozygous deletion of both FZD4 and FZD8 (*Fzd4^–/–^; Fzd8^–/–^*) results in a reduction in ureteric bud tip number as early as embryonic day (E)12.5 (Ye et al., 2011); 2 days after the commencement of murine kidney development (Lindström et al., 2018). Mice homozygous for *Looptail* (*Vangl2^*Lp/Lp*^)* or *Crash (Celsr1^*Crsh/Crsh*^)* alleles; pathogenic missense mutations in *Vangl2* and *Celsr1*, respectively, also have a reduction in ureteric bud tip number (Yates et al., 2010; Brzóska et al., 2016). Detailed phenotyping of *Celsr1^*Crsh/Crsh*^* kidneys has been performed at E13.5, by combining three-dimensional imaging and computational analysis (Short et al., 2014; Brzóska et al., 2016). In this model, the reduction in ureteric bud tip number was found to be more prominent in the caudal portion of the embryonic kidney (Brzóska et al., 2016). This phenotype worsened upon compound mutation of *Celsr1* and *Vangl2* (*Celsr1*^*Crsh*/+^; *Vangl2*^*Lp*/+^), suggesting that *Celsr1* and *Vangl2* interact during the branching of the ureteric bud, as may be the case for FZD4 and FZD8 (Ye et al., 2011). Taken together, these findings suggest that PCP proteins regulate branching morphogenesis in the kidney, although the implication of planar-polarised behaviour in this process requires the identification of molecular asymmetry of PCP proteins in ureteric bud epithelium. Moreover, an experimental model is required to test whether ureteric bud branching defects in PCP mutants are cell-autonomous, such as the specific deletion of *Vangl2* from the ureteric bud lineage using a mouse *Hoxb7-Cre* line (Derish et al., 2020).

A number of genetic experiments have also shown that molecules interacting with PCP are also involved in renal branching morphogenesis. However, the roles of these molecules appear unrelated to planar-polarised behaviours. For example, deletion of WNT11; expressed by ureteric bud tip cells in in mouse (Kispert et al., 1996) and humans (Rutledge et al., 2017), leads to reduction of murine ureteric bud tip number (Majumdar et al., 2003; O’Brien et al., 2018), but also causes a reduction in the number of cap mesenchyme cells at birth with altered polarity and disorganisation (O’Brien et al., 2018). Consequently, a reduction in the secretion of key cap mesenchyme factors involved in ureteric bud branching, such as glial-derived neurotrophic factor (GDNF) (Majumdar et al., 2003), may be responsible for the defects in branching observed in this model. *Wnt5a^–/–^* mouse kidneys, which also have reduced ureteric bud tip number (Pietilä et al., 2016), have thickened basement membranes and alterations in laminin and type IV collagen (Pietilä et al., 2016). Therefore, the impaired branching phenotype in *Wnt5a^–/–^* kidneys could be explained by defective interactions between PCP proteins and MMPs (Williams et al., 2012; Dohn et al., 2013; Jessen and Jessen, 2017); resulting in dysregulation of ECM turnover and defective ureteric bud architecture, however this hypothesis requires further exploration. Ureteric bud branching defects in mouse are also observed upon deletion of *Fat4* (Mao et al., 2011, 2015) and *Dchs1* (Mao et al., 2011; Bagherie-Lachidan et al., 2015); mammalian homologues for Ft and Ds, respectively. However, the precise role of FAT4 and DCHS1 in ureteric bud branching is not yet clear, as neither protein is localised to the ureteric bud (Mao et al., 2011, 2015; Bagherie-Lachidan et al., 2015) and these models exhibit other renal anomalies such as duplex kidney and alterations of GDNF signalling (Mao et al., 2011, 2015; Bagherie-Lachidan et al., 2015; Zhang et al., 2019).

### Planar Cell Polarity During Renal Tubular Morphogenesis

During nephron development, termed nephrogenesis, a subset of cap mesenchymal cells differentiates into epithelium whilst undergoing a series of complex morphological changes. In early nephrogenesis, epithelial cell vesicles differentiate from the cap mesenchyme, before progressing through “comma-shaped body” and “S-shaped body” stages; both precursory to the nephron (Oxburgh, 2018). This process of new nephron generation continues after birth; ceasing within the first postnatal week (Hartman et al., 2007). Based on knockout and knockdown experiments in frog and mouse, it is unclear whether the disruption of PCP proteins has significant consequence on the early stages of nephrogenesis. For example, the expression of early markers of nephron development, *Lim1* and *Hnf1*β, remain unchanged after depletion of *Daam1* by morpholino injection into eight-cell stage frog embryos (Miller et al., 2011). Moreover, all precursory stages of nephron development described above are present in *Vangl2*-, *Celsr1-*, and *Wnt11-*deficient mouse embryonic kidneys (Yates et al., 2010; Brzóska et al., 2016; O’Brien et al., 2018). Conversely, *Wnt11* mutants have over-representation of renal vesicles and reduced expression of genes involved in nephron progenitor maintenance, including *Six2* (Kobayashi et al., 2008) and *Eya1* (Xu et al., 2014), but whether this phenotype occurs due to dysregulated planar-polarised behaviours remains to be ascertained.

During late prenatal stages of mouse gestation, S-shaped bodies narrow and elongate. At these stages, the multiple differentiated segments of the nephron are detectable, including proximal tubular and collecting duct epithelium (Combes et al., 2019). At late embryonic and early postnatal stages, maturing murine renal tubules express CELSR1, VANGL1, VANGL2, FZD3, FZD4, FZD6, and FZD8 proteins (Yates et al., 2010; Babayeva et al., 2011; Ye et al., 2011; Kunimoto et al., 2017). Recent evidence also indicates the asymmetric localisation of VANGL and FZD proteins on opposing cell-cell boundaries of collecting duct and proximal tubule cells; an asymmetry that is lost in *Vangl2^*Lp/Lp*^* and *Fzd3^–/–^*; *Fzd6^–/–^* kidneys (Kunimoto et al., 2017), indicative of planar-polarised behaviour during tubule formation. Subsequent knockout studies have indicated that loss of PCP proteins results in defects in renal tubule morphogenesis. Administering an inducible dominant-negative form of DVL2 in frog embryos resulted in renal tubular dilation (Lienkamp et al., 2012). Similarly, *Vangl2^*Lp/Lp*^*, *Fzd3^–/–^*; *Fzd6^–/–^*, *Celsr1^*Crsh/Crsh*^* mouse embryos, or mice deficient of PCP-associated WNT ligands, exhibit dilation of either proximal tubules, collecting ducts or both (Karner et al., 2009; Yates et al., 2010; Babayeva et al., 2013; Brzóska et al., 2016; Kunimoto et al., 2017). When *Vangl1* or *Vangl2* are knocked out specifically in the ureteric bud or collecting duct lineages tubular dilations are replicated (Kunimoto et al., 2017; Derish et al., 2020), suggesting that these PCP proteins cell-autonomously regulate tubular diameter (Table 1). An alternative possibility is that the tubular dilation observed upon perturbation of PCP signalling in the developing kidney may be a consequence of intra-tubular pressure, as dilation of the tubules and collecting system is a phenotype consistent with other genetic models of functional obstruction (Airik et al., 2006; Caubit et al., 2008).

**FIGURE 2:**
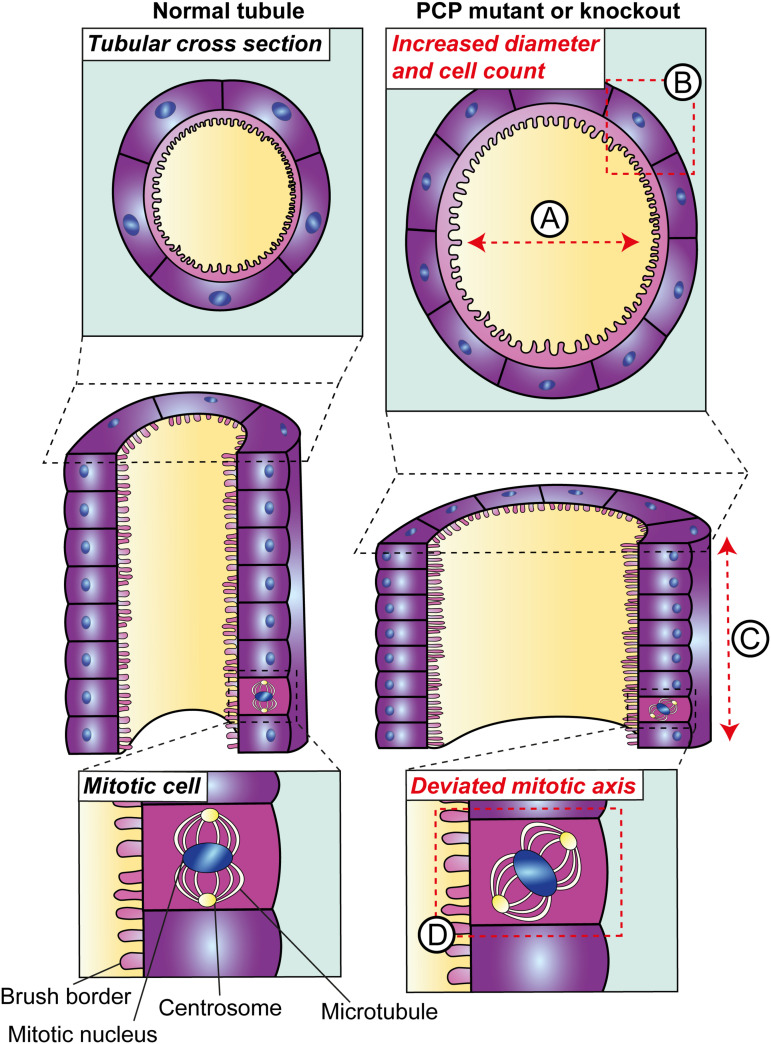
Planar cell polarity in tubular morphogenesis. Deletion of core PCP proteins from vertebrates results in defects in tubular morphogenesis during development. PCP mutant tubules are dilated **(A)**; a phenotype which may occur due to defects in two planar-polarised behaviours. The first is convergent extension, defects in which have been inferred based on the increased number of epithelial cells per tubular cross-section **(B)** and consequently a reduction in tubular elongation **(C)**. The second planar-polarised behaviour which is impaired in these models is the orientation of cell division and, in kidneys with disruption of core PCP components, this deviates from the plane of the tubule **(D)**.

Disruption of two planar-polarised behaviours are thought to underpin the tubular defects observed in PCP mutants (Figure 2). The first of these is convergent extension, which is an essential process in the narrowing and elongation of renal tubules during kidney development. Studies in embryonic mice and frogs have shown that cells of the developing renal tubular epithelium are configured in “rosettes,” formed by the contraction of linked edges of epithelial cells (Lienkamp et al., 2012). Similar epithelial rosettes are also observed in extension of the germ-band in fruit fly where they facilitate convergent extension (Blankenship et al., 2006). The resolution of these rosettes is proposed to reposition cells and facilitate tissue elongation and narrowing in a PCP-dependent process (Lienkamp et al., 2012). As evidence for this in the kidney, rosette formation in renal tubular epithelium is impaired upon inhibition of DVL2 in frog embryos (Lienkamp et al., 2012). The failure of rosette formation in the developing kidney due to PCP protein disruption has not been demonstrated in mouse models *per se.* However mouse renal tubules deficient in PCP proteins possess an increased number of epithelial cells in cross-section (Karner et al., 2009; Kunimoto et al., 2017; Derish et al., 2020). This defect has been interpreted to result from defective convergent extension movements and tubular shortening and is therefore taken as evidence for the impairment of planar-polarised behaviour.

The second planar-polarised behaviour governing tubular morphogenesis is the orientation of cell division. Toward the end of murine gestation and within the first few postnatal days; the orientation of mitotic spindles in proliferating tubular epithelial cells is largely parallel to the axis of the tubule (Fischer et al., 2006; Brzóska et al., 2016). Conversely, global knockout of *Celsr1* (Brzóska et al., 2016) or conditional knockout of *Vangl1* and *Vangl2* in tubules using *Cre* recombinase driven by cadherin 16 (CDH16, also known as kidney-specific protein) expression (Kunimoto et al., 2017) at these stages causes the orientation of dividing tubular epithelial nuclei to deviate. An understanding of the temporal relationship between defective convergent extension, misorientation of cell division and increase tubular diameter and length would be considerably enhanced by recent developments in live imaging of epithelia (Akram et al., 2019).

### Planar Cell Polarity Proteins in Podocyte Maturation

Alongside the glomerular capillary endothelium and basement membrane, podocytes are a critical component of the kidney filtration barrier. During renal organogenesis, precursors in the proximal aspect of the S-shaped body commit to a podocyte lineage (Yoshimura and Nishinakamura, 2019). From a simple columnar epithelium, these precursors differentiate, forming cells with a specialised morphology featuring cellular extensions, called foot processes, which encircle the capillary wall. Foot processes sit in apposition of each other at slit diaphragms; the main size-selective barrier in the glomerulus, preventing egress of macromolecules such as albumin into the urine. The diverse morphological changes that occur to podocytes over the course of their development (Schell and Huber, 2017) and the requirement of foot process apposition for slit diaphragm function has prompted the investigations into the expression of PCP proteins and their roles in podocyte maturation. Podocytes cultured from mouse or human express transcripts for PCP core proteins including DVL2, VANGL1, VANGL2 and CELSR1, with the latter two proteins detectable in podocytes during development of the rodent glomerulus *in vivo* (Yates et al., 2010; Babayeva et al., 2011). In the newborn rat kidney, electron microscopy and immunogold staining has demonstrated SCRIB at cell-cell contacts between immature podocytes. Conversely, in the adult rat SCRIB is localised to foot processes and the slit diaphragm (Hartleben et al., 2012). Similarly, VANGL2 has been detected at the basal aspect of developing podocytes using immunohistochemistry of the E17.5 mouse kidney, but is only weakly expressed in mature podocytes of the adult kidney (Babayeva et al., 2013).

Perturbation of PCP proteins in mouse results in glomerular defects during kidney organogenesis (Table 1). For example, *Vangl2^*Lp/Lp*^* and *Celsr1*^*Crsh*/+^/*Vangl2*^*Lp*/+^ embryonic kidneys possess a higher proportion of underdeveloped glomeruli with smaller glomerular tufts and fewer capillary loops (Yates et al., 2010; Babayeva et al., 2013; Brzóska et al., 2016). Despite a reduction of proliferating glomerular cells in *Vangl2^*Lp/Lp*^* kidneys (Yates et al., 2010), no changes in podocyte number were detected in these models. Two recent studies have refined this work by generating podocyte-specific knockout of VANGL2 using a podocin (*NPHS2)-Cre* mouse line. Both studies exploiting this model identified underdeveloped glomeruli (Rocque et al., 2015; Papakrivopoulou et al., 2018). These conditional knockout experiments indicate that VANGL2 may have a role in podocyte maturation. However, the downregulation of VANGL2 expression in adult podocytes (Babayeva et al., 2013) suggests that this role may be no longer required in fully developed podocytes. Only one of the two studies reported a subtle reduction in podocyte number which persisted into adulthood (Rocque et al., 2015); a discrepancy which could be explained by differences in quantification methods. Alternatively, the study reporting reduced podocyte number included a loss-of-function allele of *Vangl2* within their experimental cross (Rocque et al., 2015), and so discrepancy in podocyte count could result from variable efficiency of VANGL2 deletion. Whether glomerular defects in these mouse models arise from planar-polarised behaviours is not clear, as the asymmetry of expression of VANGL2 other PCP proteins has not been shown in podocytes or other glomerular cell types. Moreover, there are inconsistency between models as a glomerular phenotype is not observed in *Celsr1^*Crsh/Crsh*^* kidneys (Brzóska et al., 2016) or upon *NPHS2-Cre* mediated knockdown of *Scrib* (Hartleben et al., 2012).

Rather than orchestrating planar-polarised behaviours, evidence from mouse studies and from *in vitro* investigations point to a potential role for VANGL2 in the regulation of cytoskeletal rearrangements during podocyte development. In mouse and human podocyte lines a loss of cortical actin stress fibres is seen upon knockdown of VANGL2 using small interfering (si)RNA technology (Babayeva et al., 2011). Co-immunoprecipitation studies of HEK293 cells demonstrate VANGL2 forms a ternary complex with membrane-associated guanylate kinase inverted 2 and nephrin (Babayeva et al., 2011); both proteins of the slit diaphragm which are critical for glomerular function (Balbas et al., 2014; Li et al., 2015). In HEK293 cells, siRNA-mediated knockdown of VANGL2 reduces the internalisation and endocytosis of nephrin (Babayeva et al., 2013). These results should be interpreted with caution as they have not been reported in cultured podocytes. However, they indicate alternative functions of VANGL2 in podocytes, for example, the regulation of nephrin to facilitate cytoskeletal reorganisation in podocytes (Zhu et al., 2008). In these studies, stimulation of PCP signalling (Qian et al., 2007) by treating cells with WNT5A increased number of cortical stress fibres per cell (Babayeva et al., 2011) and increased internalisation and endocytosis of nephrin (Babayeva et al., 2013). Thus, the relationship between planar-polarised behaviours and the regulation of cytoskeletal rearrangements during podocyte maturation requires further investigation.

## The Involvement of Planar Cell Polarity in Renal Diseases

### Associations Between Variants of Planar Cell Polarity-Encoding Genes and Human Kidney Malformations

The complex renal phenotypes observed when PCP proteins are mutated or knocked down in murine models have prompted investigation into whether these proteins play a role in congenital anomalies of the kidney and urinary tract (CAKUT). CAKUT encompasses a diverse range of pathologies, including renal agenesis, fused or ectopic kidneys and vesicoureteral reflux which manifest from a combination of genetic, environmental and epigenetic risk factors (Nicolaou et al., 2015). In addition to renal phenotypes described above, deletion of PCP proteins in mice results in complete failure of the neural tube; precursory to the central nervous system, to close (Murdoch et al., 2014; Galea et al., 2018). Variants in PCP protein-encoding genes in humans have also been associated with a range of neural tube defects (Juriloff and Harris, 2012), including the most common form: spina bifida. Recent genetic screens have identified that such variants perturb planar-polarised behaviours in fruit fly (Humphries et al., 2020).

There is a long-documented association between neural tube defects and CAKUT (Roberts, 1961). These two developmental anomalies share a common link in their association with PCP protein-encoding genetic variants. In an analysis of patients from the California Birth Monitoring Program, 13 patients were identified with spina bifida and heterozygous variants in *CELSR1*, five of which had renal tract malformations detected by ultrasound in the fetal or immediate postnatal period (Brzóska et al., 2016). In this study, one patient had unilateral renal agenesis with hydronephrosis of the remaining kidney. An additional three displayed bilateral hydronephrosis and the remaining patient was diagnosed with unilateral hydronephrosis. Though the total number of cases in the patient cohort is small, this represents the first study to identify genetic variants encoding a PCP protein in individuals with both neural tube defects and CAKUT. These findings encourage larger-scale investigations to examine if PCP protein-encoding genetic variants are found in patients with neural tube defects and CAKUT.

### The Relationship Between Planar Cell Polarity and Polycystic Kidney Disease

The most common form of polycystic kidney disease (PKD) is inherited in an autosomal dominant (AD) manner and occurs due to mutations in *PKD1* and *PKD2*, encoding polycystin 1 and 2, respectively. In comparison, autosomal recessive (AR)PKD arises most commonly from mutations in *PKHD1*, encoding fibrocystin. The prevailing hypothesis in the mechanism of cystogenesis in PKD is centred around the primary cilium. PKD1, PKD2 and PKDHD1 are all expressed in primary cilia of renal tubular epithelial cells and facilitate mechanosensation and Ca^2+^ influx in response to fluid flow (Nauli et al., 2003; Ward et al., 2003). Mutation of the genes encoding these ciliary proteins lead to fluid filled epithelial cysts, which expand and occupy the renal parenchyma, eventually causing end-stage kidney disease (ESKD) (Bergmann et al., 2018). There is considerable intrafamilial variability in disease severity of both ADPKD (Lanktree et al., 2019) and ARPKD (Bergmann et al., 2005). Thus, independent of the causative mutation, there are likely unidentified genetic and environmental factors that underlie the discordance in clinical manifestation and disease severity in patients with PKD (Rossetti and Harris, 2007; Leonhard et al., 2016).

**FIGURE 3:**
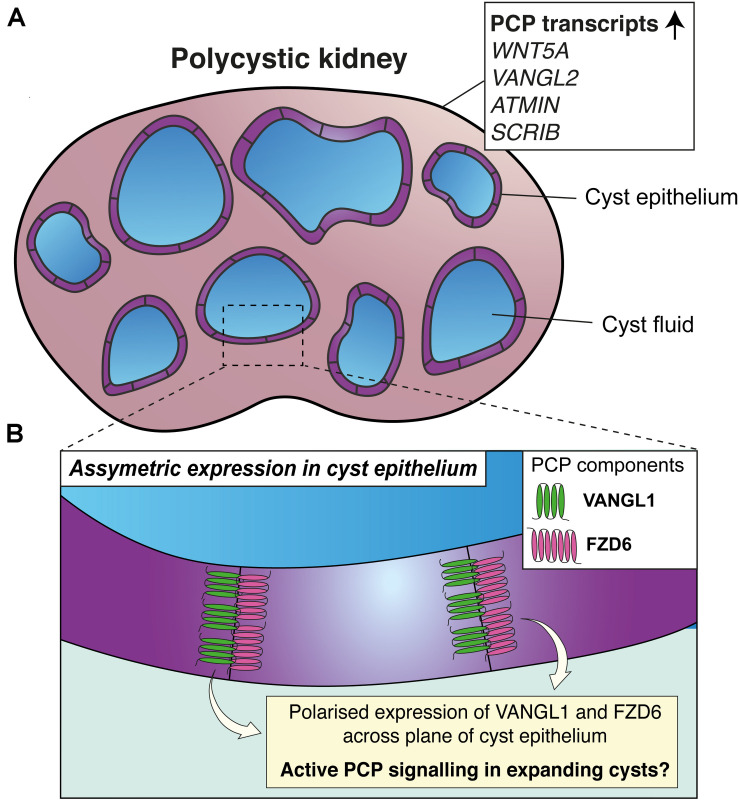
Planar cell polarity and polycystic kidney disease. **(A)** Upregulation of transcripts of proteins involved in PCP (shown in the white box) have been detected in kidney lysates from paediatric patients with autosomal recessive polycystic kidney disease (PKD). **(B)** In mouse models of PKD, opposing epithelial cells lining cysts express VANGL1 and FZD6; suggestive that PCP may be active during cyst expansion.

Some of the phenotypes that occur when core PCP proteins are deleted from mouse, such as tubular dilation and defective oriented cell division, are also observed in murine models of PKD. For example, dilation of tubules can be histologically identified during embryonic development in different mouse models of PKD. These models include the orthologous *Pkd1^*RC/RC*^* mouse (Hopp et al., 2012; Jafree et al., 2019); mimicking a human disease variant observed in patients with ADPKD (pR3277C) and resulting in slow progressive cystic disease (Hopp et al., 2012). Tubular dilation can also be observed prenatally in the non-orthologous *Cys1^*cpk/cpk*^* mouse (Avner et al., 1988), which phenocopies ARPKD pathology. Similarly, postnatal deletion of *Pkd1* from distal tubular segments of 1 week old mice, using an inducible *Mx1-Cre* allele, results in focal cystic disease (Takakura et al., 2008). In this model, average distal tubular circumference increases significantly 1 week after inactivation of *Pkd1* compared to age-matched controls (Luyten et al., 2010). In the same experiment, there was a shift in the orientation of dividing tubular epithelial cells away from the axis of the tubules (Luyten et al., 2010). Defective oriented cell division was also identified in the *pck* rat model of ARPKD (Fischer et al., 2006), which develops cystic renal disease by 3 weeks of age due to decreased expression of *Pkhd1*. Complementing these findings, kidney lysates from paediatric patients with ARPKD and ESKD contain an increased expression of PCP protein-encoding transcripts, including *WNT5A, VANGL2, ATMIN*, and *SCRIB*, compared to age-matched controls (Figure 3; Richards et al., 2019).

Several lines of evidence from knockout studies in mice suggest that disruption of core PCP proteins does not cause the initiation of cysts in PKD. Firstly, loss of function of VANGL1, VANGL2, CELSR1, or compound loss of FZD3 and FZD6 does not cause a cystic phenotype in mouse kidneys (Yates et al., 2010; Brzóska et al., 2016; Kunimoto et al., 2017; Derish et al., 2020), albeit some of these studies only examined kidneys at embryonic timepoints. Secondly, though defective orientation of cell division has been observed in rodent models of PKD (Fischer et al., 2006; Luyten et al., 2010), a non-cystic mouse model, in which the fourth exon of *Pkhd1* is disrupted in homozygosity, features defective orientation of cell division and also an increased number of nuclei in transverse sections of collecting duct tubules (Nishio et al., 2010). Moreover, upon deletion of *Pkd1* or *Pkd2* in murine collecting ducts, defective orientation of cell division has been identified to follow tubular dilation (Nishio et al., 2010). Collectively, these findings provide evidence that neither PCP proteins nor planar-polarised behaviours are responsible for initiating cyst formation in PKD.

To better understand the relationship between planar-polarised behaviours and PKD, one study partially ablated cilia from the murine renal collecting duct, which causes cystic kidney disease (Lin et al., 2003). This model featured tubular defects of convergent extension and orientation of cell division (Kunimoto et al., 2017); mirroring the phenotypes observed upon knockout of core PCP proteins. These results led the authors to postulate that planar-polarised behaviours are independently governed by PCP signalling and the activity of primary cilia, whereas only defects in the latter are required for cyst initiation. However, in the same model, asymmetric expression of VANGL1 and FZD6 was maintained in cyst epithelial cells of postnatal mice (Figure 3). Thus, molecular asymmetry and planar polarity may be a feature of the expansion of cysts after their initiation (Kunimoto et al., 2017), though a causative or correlative association is yet to be determined.

Another area of research that requires further exploration is the role of the mammalian equivalent of the Ft-Ds-Fj module in PKD. *Fat4^–/–^* and *Dchs1^–/–^* knockout mouse kidneys possess renal cysts at birth (Saburi et al., 2008; Mao et al., 2011, 2015). Though *Fat4^–/–^* and *Dchs1^–/–^* embryos possess characteristics that could suggest defective planar-polarised behaviours, such as misorientation of stereocilia in the cochlea, widening of the neural tube and disruption of orientated cell division in renal tubular epithelium (Saburi et al., 2008; Mao et al., 2011), the asymmetric expression of these proteins in tubular epithelial cells has not been assessed. Moreover, deletion of *Fjx1*; the mammalian homologue of Fj, did not worsen the cystic phenotype in mice with postnatal deletion of *Pkd1* in collecting ducts (Formica et al., 2019). Compound loss of *Fat4* with *Vangl2* in mice, however, does enhance the cystic phenotype compared to *Fat4* loss alone (Saburi et al., 2008), and raises the question as to whether FAT4 interacts with core PCP proteins to modulate cyst expansion in PKD.

### Planar Cell Polarity Proteins as Novel Players in Glomerular Disease

Morphological changes to podocytes, including the retraction and simplification of foot processes, is a common feature of many forms of glomerular injury and is underpinned by changes to cytoskeletal architecture (Schell and Huber, 2017). Experimental evidence indicating that VANGL2 regulates cytoskeletal architecture of maturing podocytes has therefore raised interest in the potential functions of this protein, or other PCP proteins, in podocyte pathology and glomerular disease. Accordingly, core PCP proteins or their transcripts have been shown to be upregulated in murine and human glomerular disease. One such mouse model of glomerular disease is the nephrotoxic nephritis (NTN) model, in which animals are pre-immunised with sheep immunoglobulin before nephritis is induced by nephrotoxic globulin. In NTN mice, progressive irreversible glomerular damage occurs, accompanied by the upregulation of glomerular transcripts for *Vangl2, Dvl2, Fzd3, Fzd6*, and *Pk1* (Rocque et al., 2015). The NTN model mirrors human rapidly progressive glomerulonephritis (RPGN) (Pippin et al., 2009), and recently, transcripts for *VANGL1, VANGL2, CELSR2, DVL2, DVL3, PK1, and PK2* were found to be significantly upregulated in two independents cohorts of RPGN biopsies compared to living kidney donor controls (Papakrivopoulou et al., 2018). Changes to these transcripts were not observed in biopsies of patients with minimal change disease compared to controls (Papakrivopoulou et al., 2018).

To begin to understand if PCP proteins have functional relevance in glomerular disease, two studies have specifically deleted VANGL2 from podocytes in mouse, which in adulthood, results in no discernible changes to the ultrastructure of the filtration barrier or significant impairment of renal function (Rocque et al., 2015; Papakrivopoulou et al., 2018). In both studies, however, it was found that downregulation of VANGL2 in podocytes enhanced the severity of NTN, with increased levels of urinary albumin and more glomerulosclerosis compared with wild-type controls (Rocque et al., 2015; Papakrivopoulou et al., 2018).

**FIGURE 4:**
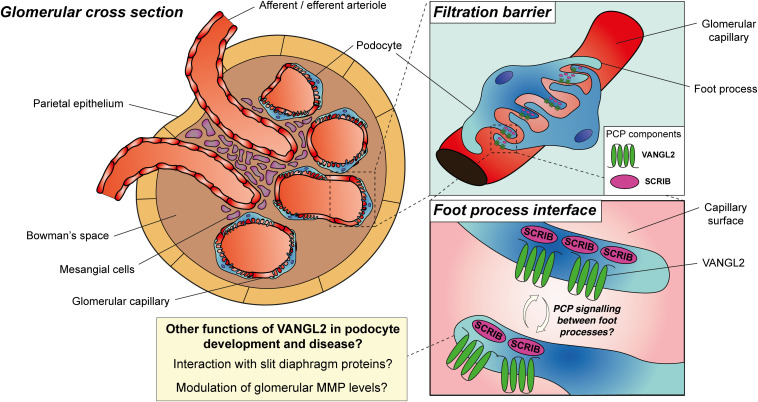
Planar cell polarity proteins in podocyte development and disease. During glomerular development, VANGL2 is expressed on the basal aspect of podocytes at the foot process interface, is downregulated in maturity and may be upregulated during glomerular injury. SCRIB expression persists in the basal aspect of podocytes and in podocyte foot processes during their maturation. PCP may be a feature of developing or diseased podocytes, facilitated by asymmetric expression of PCP proteins between interdigitating foot processes and facilitating planar-polarised behaviours. Alternatively, there is evidence for alternative roles of PCP proteins in podocyte development and disease, including their interaction with slit diaphragm proteins to regulate cytoskeletal architecture or the modulation of glomerular matrix metalloproteinase (MMP) levels.

There are several potential explanations for the increase in severity upon knockdown of podocyte VANGL2 in the NTN mouse model. Neither study related the increase in severity to changes in planar-polarised behaviours *per se*. An attractive possibility is that core PCP proteins, such as VANGL2 or FZD, are upregulated in injured podocytes and are expressed on opposing foot process interfaces to facilitate planar-polarised behaviours (Figure 4); a molecular asymmetry that is yet to be established. Against this hypothesis, the severity of doxorubicin-induced nephropathy in mice, which mimics focal segmental glomerulosclerosis in humans (Pippin et al., 2009), did not change upon podocyte-specific knockdown of the PCP-interacting protein SCRIB (Hartleben et al., 2012). Another explanation for the increase in severity of NTN when VANGL2 is deleted from podocytes is the regulation of podocyte cytoskeletal architecture by VANGL2. As VANGL2 may interact with slit diaphragm proteins such as nephrin and MAGI2 (Babayeva et al., 2011), its loss could impair the ability of podocytes to respond to injury (Schell and Huber, 2017). However, changes in cytoskeletal patterning of podocytes were not examined in either study. Other potential contributions include the loss of average podocyte number per glomerulus observed upon podocyte-specific *Vangl2* deletion, which may exacerbate the injury induced by NTN compared to control mice (Rocque et al., 2015). A potential candidate underlying the more severe glomerular injury upon podocyte-specific deletion of *Vangl2* is MMP9; the expression of which increases upon deletion of *Vangl2* from podocytes *in vitro* and *in vivo* (Papakrivopoulou et al., 2018). MMP9 is upregulated both in NTN and in lipopolysaccharide-mediated reversible glomerular injury in mice (Papakrivopoulou et al., 2018) and has previously been implicated in the progression of murine experimental RPGN through its fibrinolytic and immunomodulatory activity (Lelongt et al., 2001; Kluger et al., 2013).

## Future Directions

A substantial body of evidence implicates PCP proteins, either acting through planar-polarised behaviours or *via* alternative mechanisms, in eliciting a variety of roles during normal and abnormal kidney development, polycystic kidney disease and glomerular injury. However, several unanswered questions and possibilities still remain. From knockout and knockdown experiments in animal models, it is becoming increasingly clear that planar-polarised behaviours underpin tubular morphogenesis. However, the roles of PCP proteins or planar-polarised behaviours during branching morphogenesis in the kidney, or in other organs, remains elusive. To generate hypotheses on the roles of PCP in branching morphogenesis, possible experiments include ureteric bud-specific knockdown with sophisticated three-dimensional imaging techniques, such as two-photon (Jafree et al., 2020) or light sheet microscopy (Klingberg et al., 2017), and computational frameworks to quantify features of branching (Short et al., 2014; Jafree et al., 2019). Hypotheses generated from such experiments could then be validated by leveraging improvements in live imaging of branching morphogenesis in culture (Akram et al., 2019; Menshykau et al., 2019). Understanding the role of PCP proteins during kidney organogenesis may also herald ways to manipulate or enhance kidney organoid generation, by using CRISPR-based genome editing for example, to model diseases or to develop regenerative medicine applications (Woolf, 2019).

PCP proteins have been implicated in both inherited kidney disorders such as CAKUT and PKD and in acquired kidney diseases such as glomerulonephritis. Whether and how PCP proteins modify these diseases could be teased out by screening for genetic variants in large patient cohorts (UK10K Consortium, Walter et al., 2015) and manipulating molecular candidates using *in vitro* approaches or in animal models. It is essential that such studies differentiate between planar-polarised behaviours elicited by PCP proteins or other, alternative functions of these proteins in cellular processes. For example, the role of PCP proteins in glomerular disease and whether these involve planar-polarised behaviours in podocytes or other cell types is unclear. The discrimination between planar-polarised behaviours and alternative functions of PCP proteins could be achieved using two potential strategies. Firstly, future studies should ascertain whether the molecular basis of PCP; the asymmetry of core PCP proteins such as VANGL and FZD across the two-dimensional plane of tissue (Zallen, 2007), is a feature of events such as branching morphogenesis or podocyte detachment during glomerular inflammation. Secondly, studies seeking to understand the role of PCP signalling in renal development or pathology could make use of compound heterozygous mutations for core PCP pathway components (Brzóska et al., 2016). Such “epistatic” experiments could allow the perturbation of PCP signalling without affecting alternative functions of the proteins constituting the core pathway.

There are already several early clinical trials in oncology which are testing compounds designed to manipulate PCP signalling with the aim of improving clinical outcomes in cancer (Daulat and Borg, 2017). Repurposing compounds arising from these trials for inherited and acquired forms of kidney disease, alongside a better understanding of the involvement of PCP proteins and planar-polarised behaviours in renal diseases, could yield a new treatment paradigm for nephrology.

## Author Contributions

EP and DL prepared the first draft of the manuscript. DJ prepared the figures. All authors contributed to researching the data for the article and discussion of the review content and involved in the subsequent revision and preparation of the final manuscript for submission.

## Conflict of Interest

The authors declare that the research was conducted in the absence of any commercial or financial relationships that could be construed as a potential conflict of interest.
